# Supplementary material for: Immune-Modified Glasgow Prognostic Score Predicts Therapeutic Effect of Pembrolizumab in Recurrent and Metastatic Head and Neck Cancer
Source: Cancers (Basel). 2024 Dec 3;16(23):4056. doi: 10.3390/cancers16234056 (PMC11640115; doi:10.3390/cancers16234056)
Supplement: Supplementary file 1 [file cancers-16-04056-s001.zip › Supplemental Figures.pptx]

## Slide 1
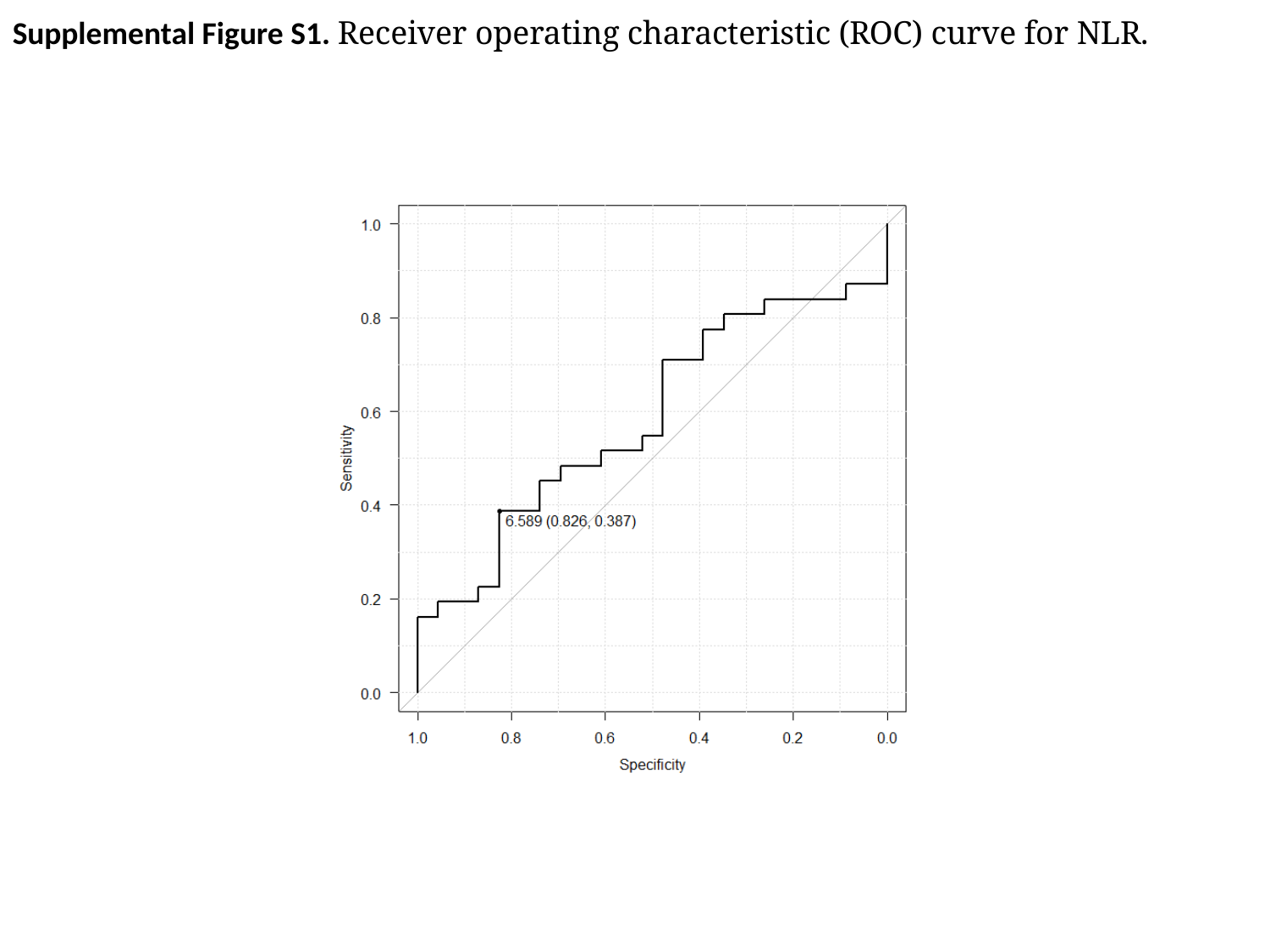

Supplemental Figure S1. Receiver operating characteristic (ROC) curve for NLR.

## Slide 2
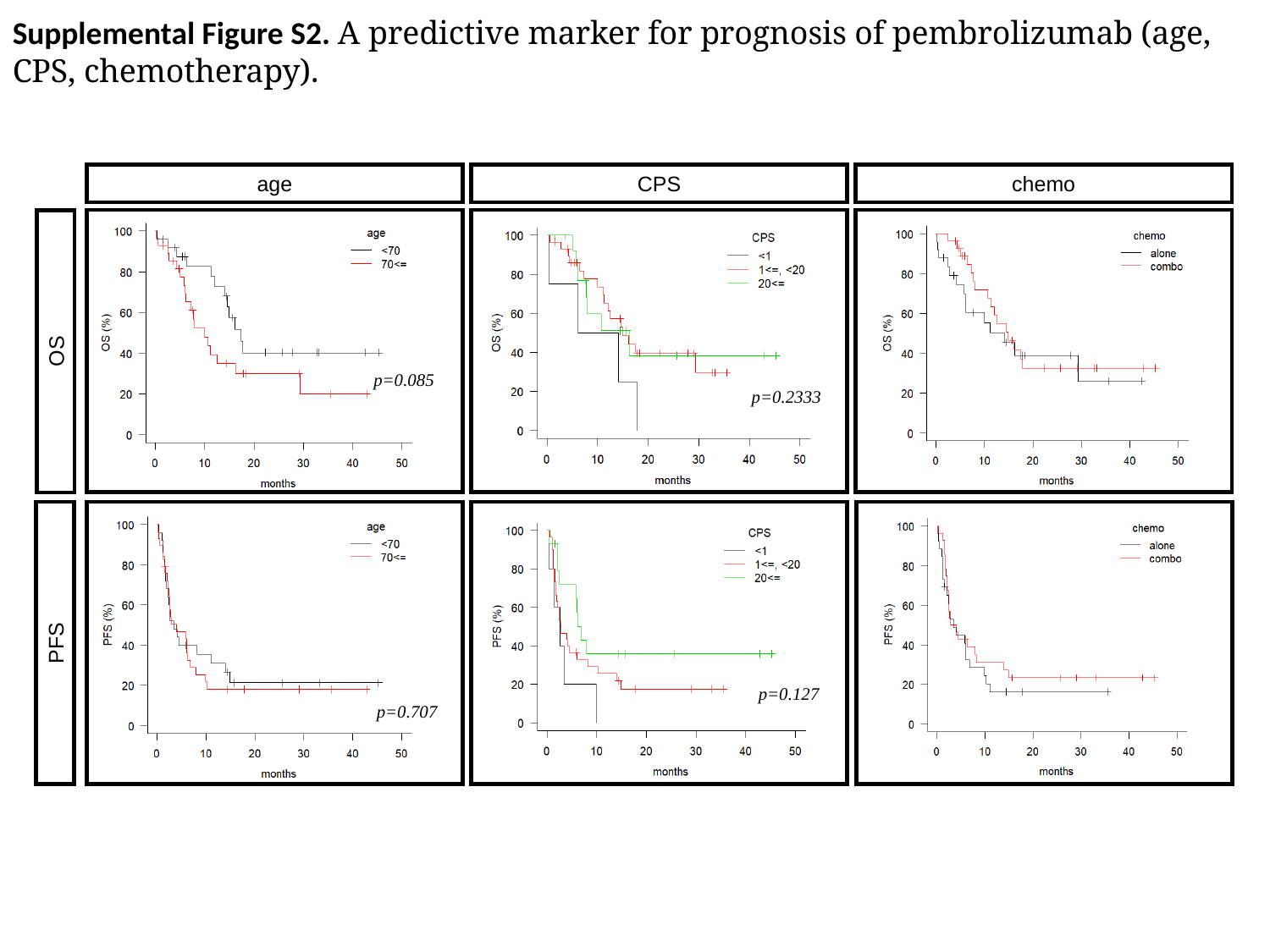

Supplemental Figure S2. A predictive marker for prognosis of pembrolizumab (age, CPS, chemotherapy).
chemo
CPS
age
OS
p=0.085
p=0.2333
PFS
p=0.127
p=0.707

## Slide 3
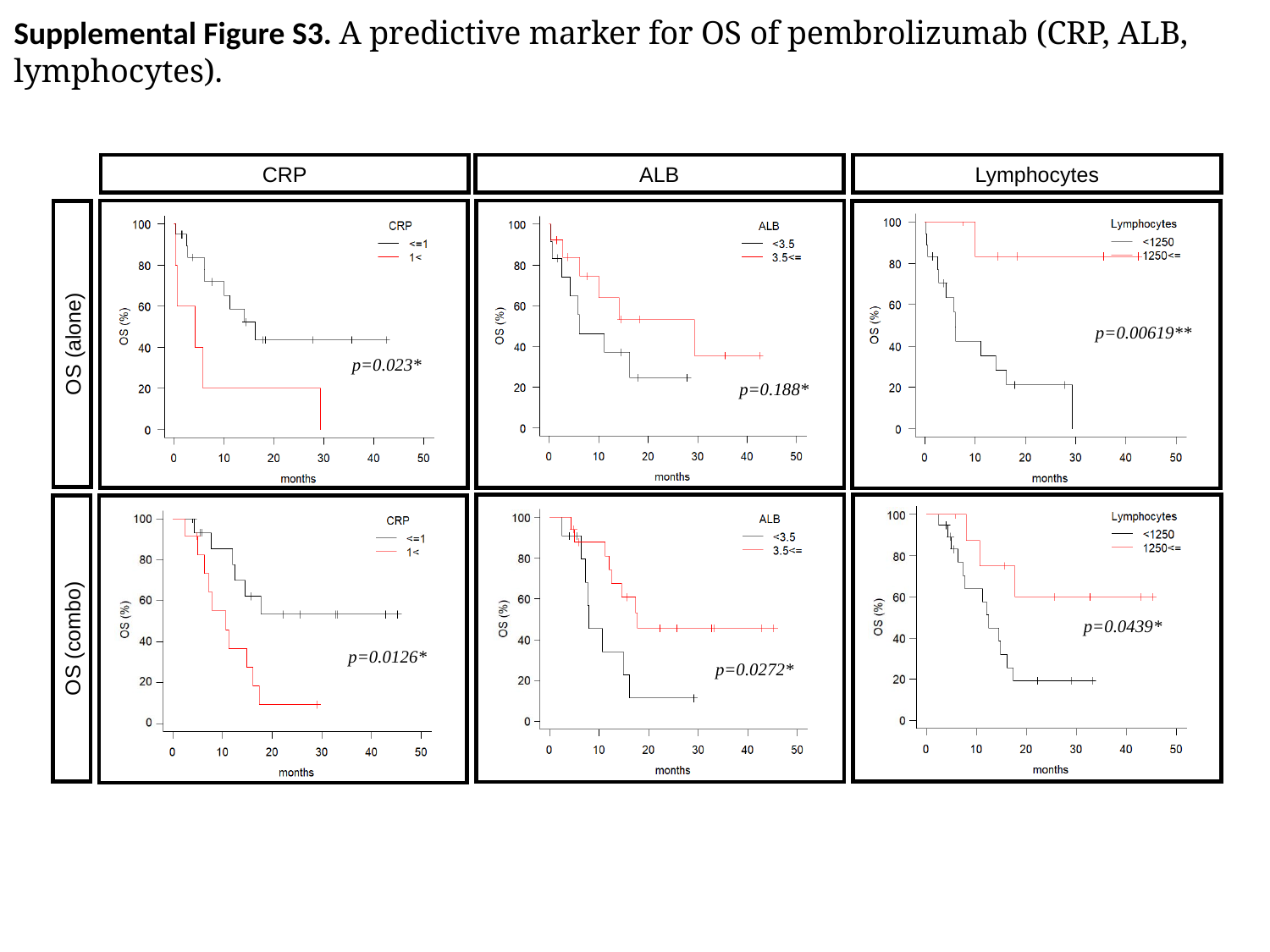

Supplemental Figure S3. A predictive marker for OS of pembrolizumab (CRP, ALB, lymphocytes).
CRP
ALB
Lymphocytes
p=0.00619**
OS (alone)
p=0.023*
p=0.188*
p=0.0439*
OS (combo)
p=0.0126*
p=0.0272*

## Slide 4
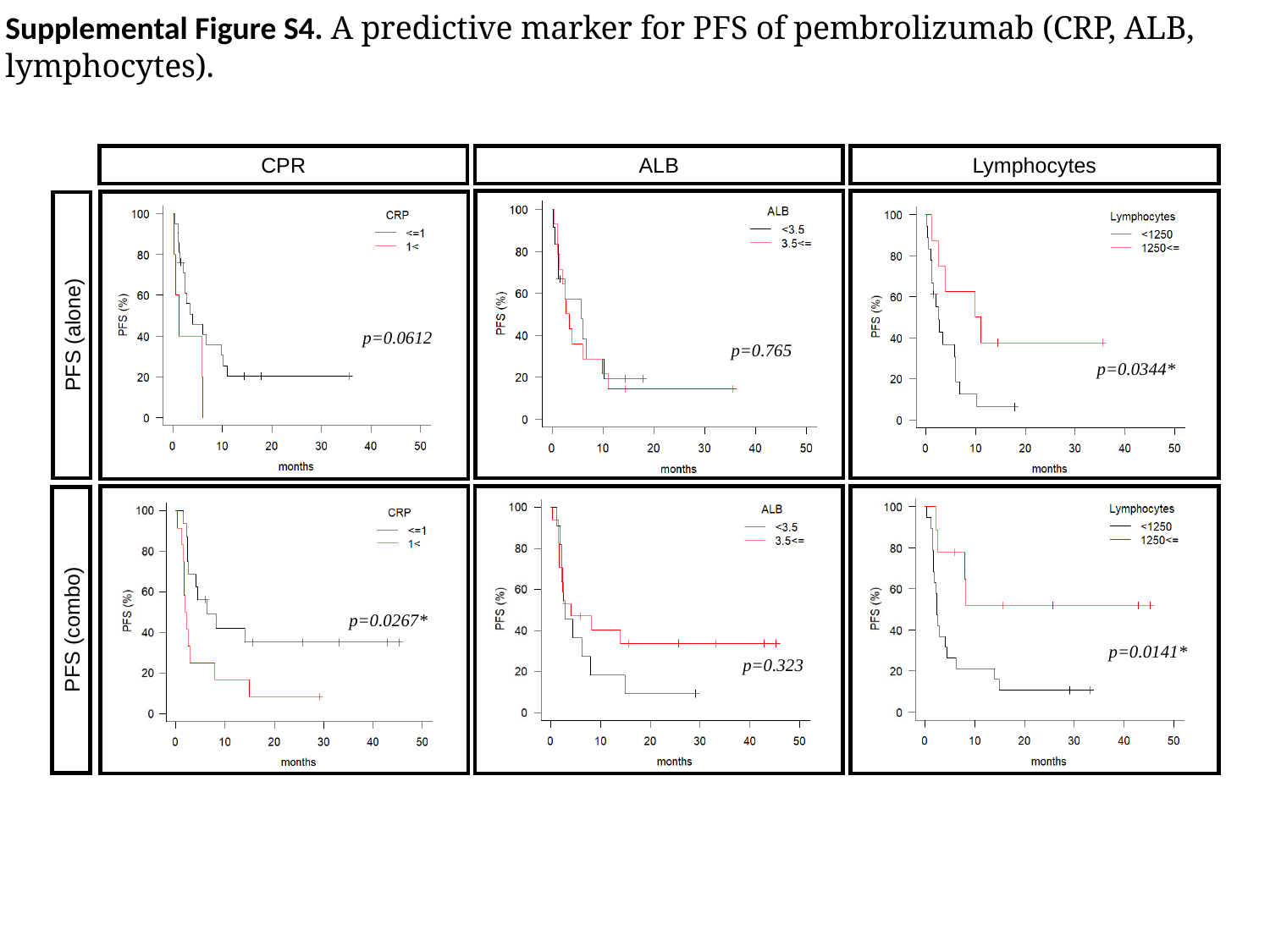

Supplemental Figure S4. A predictive marker for PFS of pembrolizumab (CRP, ALB, lymphocytes).
Lymphocytes
CPR
ALB
PFS (alone)
p=0.0612
p=0.765
p=0.0344*
p=0.0267*
PFS (combo)
p=0.0141*
p=0.323

## Slide 5
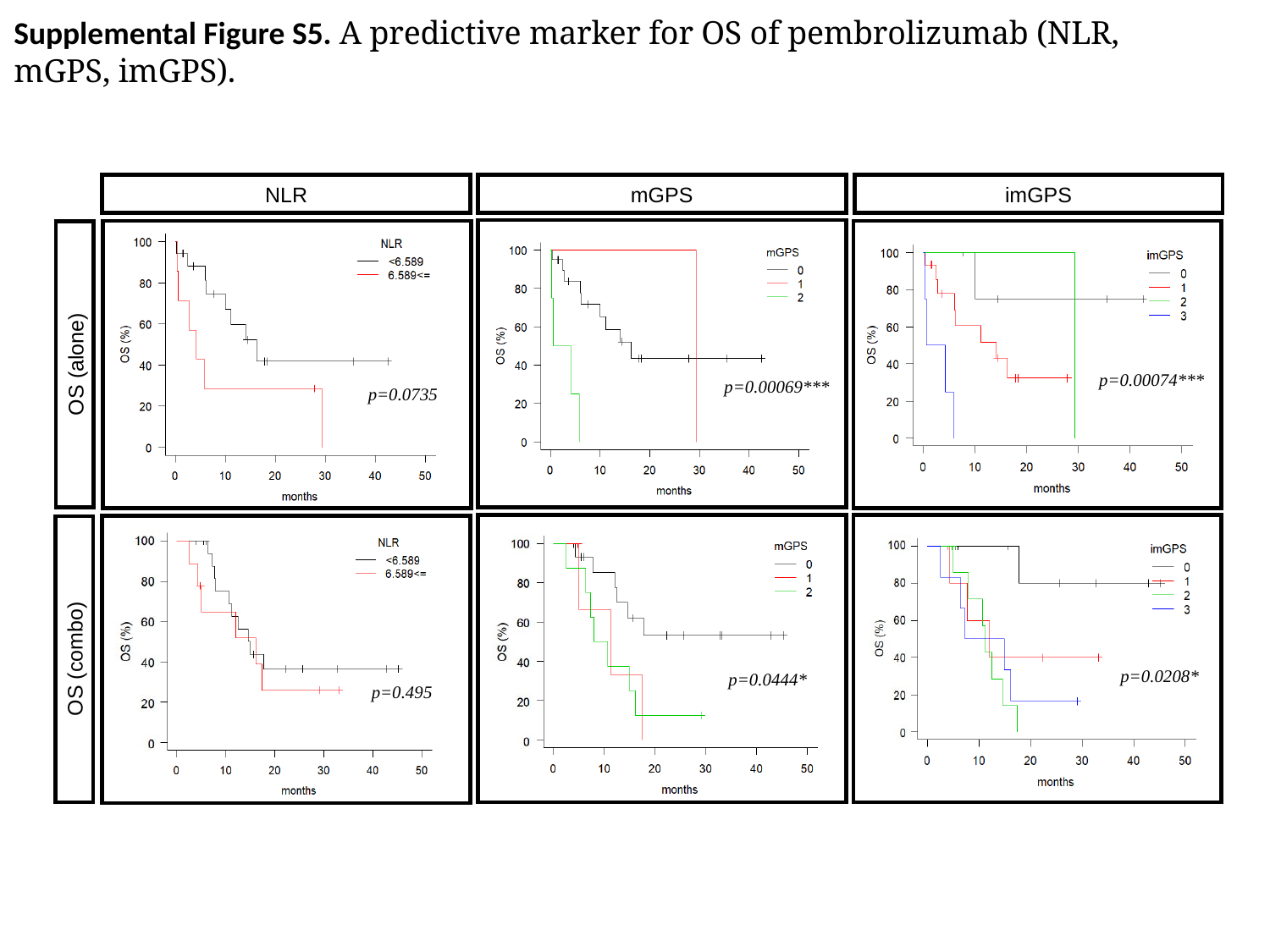

Supplemental Figure S5. A predictive marker for OS of pembrolizumab (NLR, mGPS, imGPS).
mGPS
imGPS
NLR
OS (alone)
p=0.00074***
p=0.00069***
p=0.0735
OS (combo)
p=0.0208*
p=0.0444*
p=0.495

## Slide 6
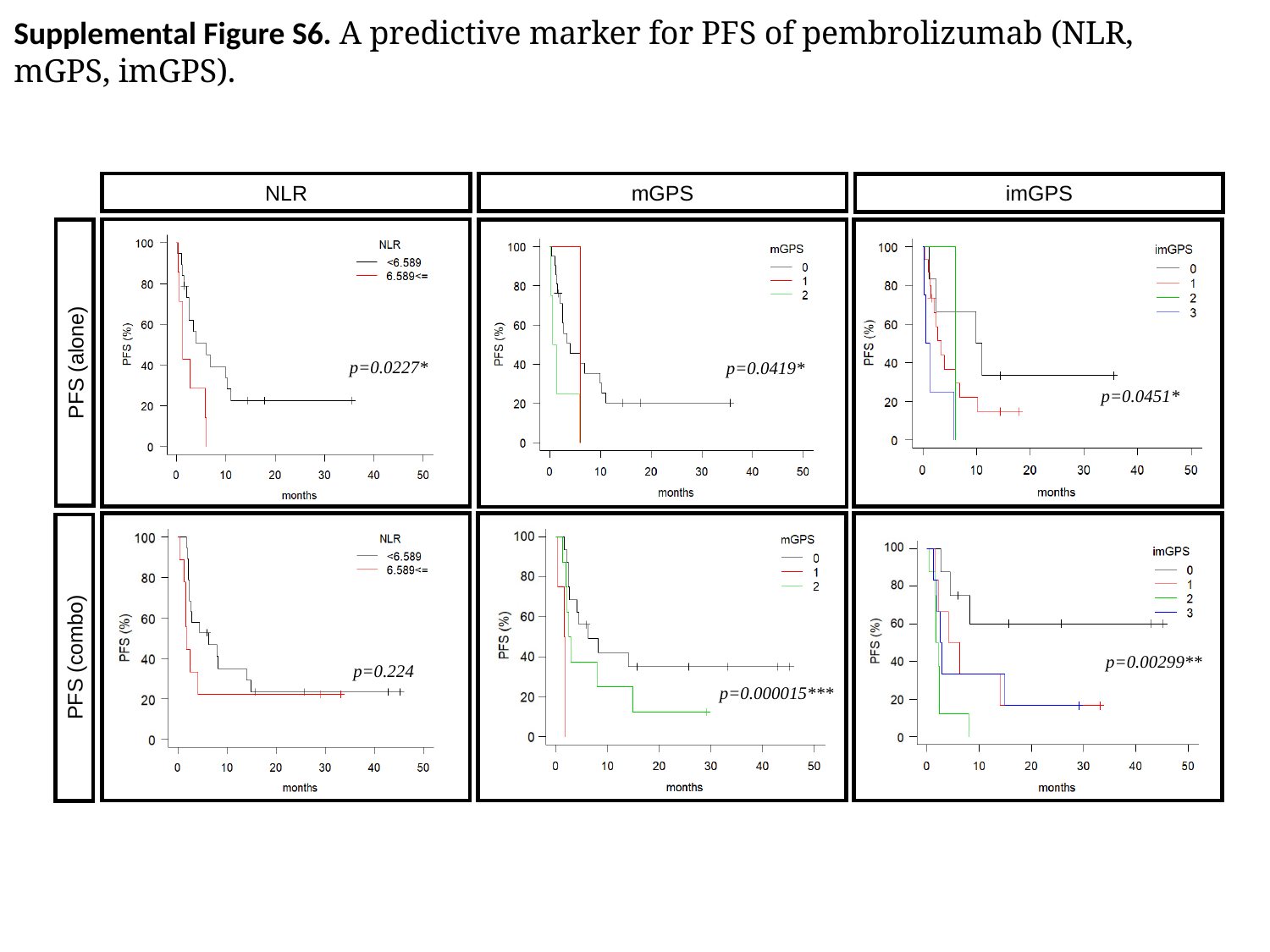

Supplemental Figure S6. A predictive marker for PFS of pembrolizumab (NLR, mGPS, imGPS).
mGPS
NLR
imGPS
PFS (alone)
p=0.0227*
p=0.0419*
p=0.0451*
PFS (combo)
p=0.00299**
p=0.224
p=0.000015***
